# Etude critique de la prise en charge de 159 personnes âgées en consultation de psychiatrie

**DOI:** 10.11604/pamj.2014.17.160.3743

**Published:** 2014-03-05

**Authors:** Jihène Ben Thabet, Yousra Ammar, Nada Charfi, Lobna Zouari, Nasreddine Zouari, Lotfi Gaha, Mohamed Maalej

**Affiliations:** 1 Service de Psychiatrie « C », CHU Hédi Chaker, Sfax, Tunisie; 2 Service de Psychiatrie, CHU Fattouma Bourguiba, Monastir, Tunisie

**Keywords:** Sujet âgé, psychiatrie, recommandations, traitement, elderly, psychiatry, recommendations, treatment

## Abstract

**Introduction:**

Le phénomène de vieillissement des populations est associé à une augmentation de la prévalence de la morbidité liée à l’âge. La prescription des psychotropes chez le sujet âgé est de plus en plus fréquente dans les institutions, les doses sont de plus en plus élevées, avec un recours fréquent à une poly pharmacothérapie. Nous nous sommes proposé de décrire les conduites thérapeutiques chez le sujet âgé consultant en psychiatrie, en vue de les confronter aux dernières recommandations en la matière.

**Méthodes:**

L’étude était de type rétrospectif et descriptif. Elle a concerné les sujets âgés d'au moins 60 ans ayant consulté pour la première fois en psychiatrie, au CHU Hédi Chaker à Sfax, en 2010 ou 2011.

**Résultats:**

Nous avons colligé 159 dossiers. L’âge moyen était de 73 ans. La démence et les troubles de l'humeur étaient les diagnostics les plus fréquents. Sur le plan thérapeutique, une poly thérapie faite d'au moins deux psychotropes de familles différentes a été prescrite pour 55,9%. Chez 60.3% des sujets, le traitement a été prescrit d'emblée à dose complète. Aucun dossier ne faisait état d'une prise en charge psychothérapeutique.

**Conclusion:**

La prise en charge des malades de notre étude n’était pas conforme aux recommandations, notamment en matière d'association médicamenteuse, de progression des doses et d'association de la psychothérapie à la pharmacothérapie. L'information des médecins et leur sensibilisation aux particularités du sujet âgé contribuerait à optimiser les soins qui leur sont prodigués, y compris en psychiatrie.

## Introduction

Le vieillissement, de par les transformations physiques, socioculturelles et familiales qu'il induit, et les nombreuses pertes qui l'accompagnent, est une période où les remaniements psychiques s'imposent pour permettre au sujet d'affronter sa situation nouvelle [1]. Le phénomène de vieillissement des populations est également associé à une augmentation de la prévalence de la morbidité liée à l’âge. Kramer [2] a annoncé il y a trente ans une « pandémie de troubles mentaux et des incapacités associées ». L'Organisation Mondiale de la Santé (OMS) a également estimé que les troubles mentaux constituaient déjà l'une des premières causes de morbidité et les trois cinquièmes de toutes les sources d'incapacités liées au vieillissement [3, 4]. La Tunisie n’échappe pas à cette réalité mondiale. Elle a connu une transition démographique avec de profonds changements de la pyramide des âges en faveur du 3ème âge [5]. La loi Tunisienne (loi 94-114) considère qu'on est une personne âgée à partir de 60 ans [6]. La prescription des psychotropes chez le sujet âgé est de plus en plus fréquente dans les institutions, les doses sont de plus en plus élevées, avec un recours fréquent à une poly pharmacothérapie [7]. Elle est caractérisée par un rapport bénéfice/risque faible. Leur usage se restreint le plus souvent au traitement symptomatique des troubles secondaires à des difficultés d'adaptation du sujet âgé à son environnement [7].

## Méthodes

L’étude était de type rétrospectif et descriptif. Elle a concerné les sujets âgés d'au moins de 60 ans, traités à titre externe, à l'unité des consultations externes de psychiatrie, au CHU Hédi Chaker à Sfax, durant les deux années 2010 et 2011. Nous avons transcrit sur une fiche, à partir des dossiers, les données sociodémographiques, les comorbidités somatiques, le(s) diagnostic(s) retenu(s), les traitements prescrits, les doses et les effets indésirables. L'analyse statistique a été réalisée par le logiciel SPSS dans sa dix-huitième version.

## Résultats

Durant les deux années concernées par l’étude (2010 et 2011), 175 sujets âgés d'au moins 60 ans ont consulté pour la première fois à l'unité des consultations externes de psychiatrie au CHU Hédi Chaker à Sfax. Ils représentaient 8.4% des nouveaux consultants en 2010 et 2011, tous âges confondus. Seize dossiers étaient inexploitables. Notre étude a finalement porté sur 159 sujets âgés d'au moins 60 ans.

### Profil sociodémographique

La moyenne d’âge de la population étudiée était de 73 ans, avec des extrêmes de 60 et 96 ans. Le sex-ratio (H/F) était de 0.83 (homme =45.3% et femme =54.7%). Sur le plan professionnel, ils étaient auparavant fonctionnaires (10%), de profession libérale (10.7%) ou journaliers (23.3%); 56% étaient sans activité professionnelle. Parmi ceux qui étaient professionnellement actifs, 34.3% l’étaient de façon régulière et 65.7% de façon irrégulière. L'activité professionnelle était encore maintenue au temps des consultations pour 3.8% des patients. Le niveau socioéconomique était bas dans 75.5% des cas, moyen (1 à 2 fois le SMIG /membre de famille) dans 22.6% cas et élevé dans 1.9% des cas.

### Antécédents personnels

Des comorbidités somatiques ont été rapportées par 68,4% des patients. Elles étaient représentées essentiellement par l'hypertension artérielle (HTA) (27.6%), le diabète (19.5%), les cardiopathies (5.7%) et l'asthme ou une broncho-pneumopathie obstructive (BPCO) (3.1%). Des antécédents psychiatriques ont été trouvés chez 27.7% des participants. Parmi eux, 88.6% avaient consulté auparavant en libre pratique et 25.6% avaient été hospitalisés au moins une fois en psychiatrie. Le taux de ceux qui ont été antérieurement mis sous monothérapie était de 65.7%: un antidépresseur (22.9%), un antipsychotique conventionnel (25.7%), un antipsychotique de seconde génération (2.9%), un tranquillisant (11.4%) et un antiépileptique thymorégulateur (2.9%). Ceux qui avaient déjà été mis sous poly thérapie représentaient 34.3% des patients.

### Trouble actuel

La démence et les troubles de l'humeur étaient les diagnostics les plus fréquemment retenus, avec des taux respectifs de 40.2% (64 patients) et 34.6% (55 patients). Les troubles de l'humeur étaient de types dépressif pour 87.3% et bipolaire pour 12,7%. Le diagnostic de maladies neurologiques diverses a été retenu chez 5.5%.

### Prise en charge thérapeutique

La monothérapie a été utilisée chez 37.8% des malades. La répartition des psychotropes était la suivante: un antidépresseur (33.3%) dont 82% d'inhibiteur sélectifs de la recapture de la sérotonine (ISRS), 12.3% d'inhibiteur de la recapture de la sérotonine et de la noradrénaline (IRSNa) et 5.7% de tricycliques, un antipsychotique conventionnel (28.3%), une benzodiazépine (21.7%), un antipsychotique de seconde génération (15.1%) et un antiépileptique thymorégulateur (1.6%). Une poly thérapie faite d'au moins deux psychotropes de familles différentes a été prescrite chez 55,9% des patients. Les associations thérapeutiques les plus fréquentes étaient: un antidépresseur et une benzodiazépine (32.5%); un antipsychotique conventionnel, une benzodiazépine et un correcteur (10.2%); un antipsychotique conventionnel, un antidépresseur et une benzodiazépine (8.9%); un antipsychotique de seconde génération, une benzodiazépine et un correcteur (8.9%).

En tout, 95 patients (59.7% de la population de l’étude) étaient sous traitement antidépresseur dont 78 (82%) sous ISRS, 12 (12.3%) sous IRSNa et 5 (5.7%) sous tricycliques. Quatre-vingt-huit patients (55.3%) étaient sous antipsychotique, dont 67.1% de type conventionnel et 32.9% de seconde génération. Soixante-cinq patients (40.8%) étaient sous benzodiazépine. Parmi les 64 sujets déments, 18 (27.9%) étaient sous traitement antidépresseur dont 77.8% sous ISRS, 16.7% sous IRSNa et 5.5% sous tricyclique. Quarante-trois patients (62.5%) étaient sous antipsychotique dont 68.9% de type conventionnel et 31.1% de seconde génération. Trente patients (46.5%) étaient sous benzodiazépine dont 25% en monothérapie. Le traitement psychotrope a été prescrit d'emblée à dose complète dans 60.3% des cas. Une moitié dose a été prescrite dans un premier temps avec une augmentation progressive des doses dans 39.7% des cas.

Dans 5% des cas, des effets indésirables ont été notés. Il s'agissait d'hypotension orthostatique pour 1.9%, de syndrome extrapyramidal pour 1.3%, de confusion mentale pour 1.3% et d'effet anti cholinergique pour 0.6%. Aucun dossier ne faisait état d'une prise en charge psychothérapeutique, toutes pathologies confondues. Dans 6.3% des cas, les patients n'avaient reçu ni traitement médicamenteux ni psychothérapie.

## Discussion

### Profil du sujet âgé consultant en psychiatrie

Selon notre étude, le sujet âgé consultant en psychiatrie avait une moyenne d’âge de 73 ans, avec un sex-ratio proche de 1. Près des 2/3 d'entre eux souffraient d'une comorbidité somatique à type d'hypertension artérielle (27.6%), de diabète (19.5%), de cardiopathie (5.7%) ou de pathologie respiratoire (asthme ou BPCO; 3.1%). Ce résultat est comparable à celui trouvé par khammassi et al. [7], dans une étude réalisée dans la région de Tunis, où la comorbidité somatique était également dominée par les pathologies vasculaire (51%), métabolique (37%) et neurologique (37%). En fait, l'association entre morbidité psychiatrique et morbidité vasculaire est une notion bien connue [7].

La fréquence élevée de la comorbidité somatique chez les sujets âgés consultant en psychiatrie atteste la nécessité que la prise en charge de tels malades soit multidisciplinaire pour bien traiter à la fois les maladies psychiatriques et les problèmes somatiques. En France, la prise en charge de tels malades est centrée à la fois sur les domaines psychiatrique, somatique et social [8].

### Diagnostic retenu

**Démence:** La démence était le trouble le plus fréquent chez nos malades (40.2%). Dans une étude faite dans les années 1990, qui a porté sur 211 cas de sujets âgés ayant consulté dans le même service de psychiatrie dans lequel notre étude a été menée, la démence représentait 26% de l'ensemble des diagnostics retenus [9]. En moins de 20 ans, la prévalence de ce diagnostic a augmenté de 50%. Il ne s'agit sans doute pas uniquement d'une amélioration des moyens de dépistage ou d'une familiarisation avec cette pathologie. Les mutations socioculturelles, avec l’évolution vers la famille nucléaire aux dépens de la famille traditionnelle élargie, ont pour corollaire une moindre tolérance vis-à-vis du sujet âgé et de sa turbulence et une demande relativement élevée de soins pour les troubles psycho-comportementaux chez les déments.

**Trouble dépressif majeur:** Le trouble dépressif majeur est l'un des troubles psychiatriques les plus fréquents chez le sujet âgé [4]. En réalité, l’âge ne constitue pas en soi un facteur de risque pour la survenue d'un syndrome dépressif. En revanche, un certain nombre de facteurs externes accompagnant habituellement le vieillissement sont péjoratifs. En effet, quelle que soit la nature de la dépression, l'influence et l'impact des facteurs biologiques, situationnels, sociaux et psychologiques seraient plus importants dans sa survenue chez les sujets âgés [10]. Dans une revue portant sur la littérature des 10 dernières années, Charney et al. [11] notent que, malgré l'existence de médicaments efficaces, la dépression du sujet âgé reste un problème de santé publique majeur. En effet, elle reste associée à une perte d'autonomie, à un déclin fonctionnel, à une baisse de la qualité de vie, à un accroissement de la mortalité lié aux comorbidités et aux suicides. Elle constitue un fardeau pour les aidants et une charge importante pour les services de santé.

**Prise en charge thérapeutique**: Parmi nos patients, 55.9% avaient reçu une poly thérapie psychotrope. Pour les patients qui avaient déjà été hospitalisés ou traités en libre pratique, le taux de ceux qui étaient sous poly thérapie psychotrope était de 34.3%. La poly thérapie psychotrope pour les malades âgés était manifestement plus fréquente à l'unité des consultations externes de psychiatrie de l’étude, comparativement aux services internes et au secteur privé. Rappelons, à ce propos, qu'il est recommandé de privilégier la monothérapie, toutes classes de médicaments confondues, surtout pour les personnes âgées [12].

En effet, la poly médication est le principal facteur de risque d'iatrogénie, dont les conséquences sont sévères chez les patients âgés. Elle diminue la qualité de l'observance et a un coût élevé. L'instauration de tout nouveau traitement doit donc être réfléchie. Les objectifs et les modalités du traitement doivent tenir compte des comorbidités et de la qualité de l'observance [13]. Les psychotropes les plus fréquemment prescrits étaient les antidépresseurs (59.7%) dont 82% d'ISRS, 12.3% d'IRSNa et 5.7% de tricycliques.

Les ISRS sont actuellement recommandés en première intention dans le traitement des états dépressifs du sujet âgé [14] alors que les antidépresseurs tricycliques sont déconseillés en raison de leur effet délétère sur les fonctions cognitives des personnes âgées surtout celles atteintes d'un état de démence [15]. Il est également recommandé d'utiliser un antidépresseur sans effet cholinergique [12]. L'association thérapeutique la plus fréquente était: un antidépresseur et une benzodiazépine (32.5%). En France, la haute autorité de santé [12] recommande, lors du traitement des épisodes dépressifs, d’éviter ou de limiter les coprescriptions, notamment à visée sédative, anxiolytique ou hypnotique.

Dans notre série, les benzodiazépines ont été prescrites chez 40.8% des patients. En France, environ 30% des personnes de plus de 65 ans consomment des benzodiazépines; elles sont 20% au Canada et en Espagne et environ 15% en Australie [16]. La prescription de benzodiazépines devrait être très limitée chez les personnes âgées. Ces médicaments présentent des effets délétères potentiels en particulier chez de telles personnes. Une étude faite par Billioti de Gage S. et al, sur 1063 patients âgés de plus de 50 ans, avait montrée que l'utilisation de benzodiazépines était associée à une augmentation d'environ 50% du risque d'apparition de démence. Ainsi, à côté des risques connus de chutes et de fractures imputables à l'usage des benzodiazépines chez les personnes âgés, des travaux de plus en plus nombreux s'accordent pour mettre en garde contre le risque de démence associé à cette utilisation [16]. L'indication des tranquillisants, et en l'occurrence des benzodiazépines, peut être justifiée, mais de façon très restreinte, dans l'anxiété [17]. Elle doit être limitée aux situations de crise ou doit être de courte durée après correction des causes: somatiques, relationnelles, psychologiques ou iatrogéniques [12].

Quatre-vingt-huit patients (55.3%) de notre population d’étude étaient sous un antipsychotique, dont 67.1% conventionnels et 32.9% de seconde génération. Pour Rigaud et al. [4], le choix de l'antipsychotique chez la personne âgée doit être guidé par deux nécessités: d'une part choisir une molécule adaptée en fonction des symptômes, d'autre part réduire les effets secondaires au minimum. Chez la personne âgée, les molécules utilisées sont les antipsychotiques conventionnels mais aussi les antipsychotiques de seconde génération, qui devraient être privilégiés du fait de leur meilleure tolérance [18]. Les données actuelles de la littérature médicale insistent sur la tolérance relative et l'efficacité de ces derniers [19, 7], prescrits à faibles doses chez le sujet âgé [20, 21], particulièrement dans les troubles du comportement des états de déficit cognitif [22] comme la maladie d'Alzheimer [23], la démence à corps de Lewy [24] et les états confusionnels en milieu hospitalier [25]. Ces nouvelles molécules semblent occasionner moins souvent que celles conventionnelles un syndrome extrapyramidal ou une hyperthermie maligne [26].

Dans notre population d’étude, nous avons eu recours à deux fois plus d'antipsychotiques conventionnels que ceux de seconde génération. Le bas niveau socio-économique et l'absence d'activité professionnelle antérieure (respectivement, 75.5% et 56% dans notre étude) étaient les deux premières causes d'absence de couverture sociale pour la plupart de nos patients, d'où le recours plus fréquent aux antipsychotiques conventionnels, seuls antipsychotiques disponibles dans les structures publiques de santé durant la période concernée par l’étude. La haute autorité de santé française [12] fait remarquer que les antipsychotiques sont actifs sur les signes psychotiques, ainsi que sur l'agitation et l'agressivité qui en découlent. Elle souligne aussi le fait que les antipsychotiques (classiques et atypiques) exposent à un risque relativement élevé de décès et d'accidents vasculaires cérébraux, et déconseille leur usage chez les personnes atteintes de maladie d'Alzheimer ou d'une maladie apparentée.

Avant toute prescription d'un antipsychotique, il est recommandé d’évaluer le rapport bénéfices/risques [12] en tenant compte: De ses effets secondaires neurologiques extra-pyramidaux (akathisie, syndrome parkinsonien, dyskinésies tardives); Du risque de: chutes, fausses routes alimentaires, sédation excessive, troubles métaboliques, hypotension orthostatique, troubles du rythme et de la conduction cardiaque; De ses éventuels effets anti-cholinergiques (risque de troubles cognitifs, de constipation, de rétention urinaire).

Il est recommandé de ne prescrire un antipsychotique qu'en cas de trouble psychotique sévère et non contrôlable autrement, après échec des autres mesures non médicamenteuses ou en cas d'urgence (danger pour le patient lui-même ou pour autrui). En cas de décision de prescription, il est recommandé de suivre les règles suivantes: Evaluer systématiquement le risque d’événements cérébro-vasculaires, cardiaques, neurologiques, cognitifs et métaboliques;Identifier, documenter et quantifier systématiquement les symptômes cibles à corriger;Choisir l'antipsychotique après une analyse individuelle des bénéfices/risques: demi-vie courte, effet anti-cholinergique faible;Informer systématiquement le patient ou l'aidant sur le rapport bénéfices/risques du traitement;Utiliser systématiquement la posologie initiale la plus basse possible, de l'ordre du quart des posologies usuelles chez l'adulte jeune, puis l'augmenter progressivement si besoin;Prescrire le traitement pour une durée très limitée;Réévaluer systématiquement, au moins toutes les semaines, la tolérance physique, neurologique et cognitive et l'efficacité symptomatique;Arrêter les antipsychotiques dès que l’état clinique le permet ou dès que les autres mesures thérapeutiques sont devenues efficaces.

Pour nos malades, le traitement a été prescrit d'emblée à dose complète dans 60.3% des cas; une moitié-dose a été prescrite au début avec une augmentation progressive des doses dans 39.7% des cas. Théoriquement, il est recommandé de démarrer à de petites doses, en augmentant prudemment par paliers, et de maintenir le traitement à la dose efficace la mieux tolérée [12]. Malgré le fait qu'il est communément admis que la psychothérapie constitue un traitement dont l'intérêt est indiscutable dans toutes les pathologies psychiatriques [12], aucun dossier ne faisait état d'une prise en charge psycho thérapeutique, ce qui témoigne d'une lacune dans la formation des praticiens, du moins ceux concernés par l’étude. On parle aussi à titre de boutade de « chimiatres » pour qualifier les psychiatres qui recourent uniquement à la chimiothérapie au dépend de la psychothérapie. Il y a aussi une raison pratique: la chimiothérapie, c'est plus rapide. Même ceux qui sont initiés aux thérapies cognitivo-comportementales (TCC) recourent plutôt aux médicaments.

Dans 6.3% des cas, les patients de l’étude n'avaient reçu ni traitement ni psychothérapie. Il s'agirait plutôt de malades mal orientés, comme en témoigne le diagnostic définitivement retenu chez 5.5% d'entre eux et qui était en rapport avec des maladies neurologiques diverses.

## Conclusion

Partout dans le monde, la prévalence des troubles psychiatriques est importante chez les personnes âgées; près de la moitié des sujets de plus de 60 ans en seraient atteints. La prescription des psychotropes chez le sujet âgé est de plus en plus fréquente avec un recours fréquent à une poly pharmacothérapie. Dans ce travail, nous nous sommes proposé de décrire les conduites thérapeutiques chez le sujet âgé consultant en psychiatrie, en vue de les confronter aux dernières recommandations en la matière. Notre étude a montré que la prise en charge du sujet âgé consultant en psychiatrie dans la région de l’étude n’était pas conforme aux dernières recommandations, notamment en matière d'association médicamenteuse, de progression posologique et d'association de la psychothérapie à la pharmacothérapie. L'information des médecins et leur sensibilisation aux particularités du sujet âgé, notamment dans le cadre de la formation continue et de l'enseignement post-universitaire (mastères de gériatrie et de gérontopsychiatrie), contribuerait à optimiser les soins prodigués aux sujets âgés, y compris en psychiatrie.
